# Efficacies of the new Paclitaxel-eluting Coroflex Please™ Stent in percutaneous coronary intervention; comparison of efficacy between Coroflex Please™ and Taxus™ (ECO-PLEASANT) trial: study rationale and design

**DOI:** 10.1186/1745-6215-10-98

**Published:** 2009-10-23

**Authors:** Jae-Bin Seo, Hui-Kyung Jeon, Kyung-Woo Park, Jong-Seon Park, Jang-Ho Bae, Sang-Wook Kim, Keon-Woong Moon, Jae-Woong Choi, Sang-Gon Lee, Woo-Young Chung, Tae-Jin Youn, Soo-Joong Kim, Doo-Il Kim, Byung-Ok Kim, Min-Su Hyon, Keum-Soo Park, Tae-Joon Cha, Hweung-Kon Hwang, Seung-Ho Hur, Hyo-Soo Kim

**Affiliations:** 1 Cardiovascular Center, Seoul National University Hospital, Seoul, Korea; 2 Uijeongbu St Mary's Hospital, Uijeongbu, Korea; 3 Yeungnam University Medical Center, Daegu, Korea; 4 Konyang University Hospital, Daejeon, Korea; 5 Chung-Ang University Medical Center, Seoul, Korea; 6 St Vincent's Hospital, Suwon, Korea; 7 Eulji General Hospital, Seoul, Korea; 8 Ulsan University Hospital, Ulsan, Korea; 9 Boramae Medical Center, Seoul, Korea; 10 Seoul National University Bundang Hospital, Sungnam, Korea; 11 Kyunghee University Medical Center, Seoul, Korea; 12 Inje University Pusan Paik Hospital, Busan, Korea; 13 Inje University Sang-gye Paik Hospital, Seoul, Korea; 14 Soon Chun Hyang University Hospital, Seoul, Korea; 15 Inha University Hospital, Incheon, Korea; 16 Kosin University Gospel Hospital, Busan, Korea; 17 Sejong General Hospital, Bucheon, Korea; 18 Keimyung University Dongsan Medical Center, Daegu, Korea

## Abstract

**Background:**

Previous randomized trials have showed the superiority of Paclitaxel-eluting stent over bare metal stent in angiographic and clinical outcomes. Coroflex Please™ stent is a newly developed drug eluting stent using the Coroflex™ stent platform combined with the drug paclitaxel contained in a polymer coating. PECOPS I trial, one-arm observational study, showed that the clinical and angiographic outcomes of Coroflex Please™ stent were within the range of those of Taxus, the 1^st ^generation paclitaxel-eluting stent (PES). However, there have been no studies directly comparing the Coroflex Please™ stent with the Taxus Liberte™ stent that is the newest version of Taxus. Therefore, prospective, randomized trial is required to demonstrate the non-inferiority of Coroflex Please™ stent compared with Taxus Liberte™ stent in a head-to-head manner.

**Methods:**

In the comparison of Efficacy between COroflex PLEASe™ ANd Taxus™ stent(ECO-PLEASANT) trial, approximately 900 patients are being prospectively and randomly assigned to the either type of Coroflex Please™ stent and Taxus Liberte™ stent via web-based randomization. The primary endpoint is clinically driven target vessel revascularization at 9 months. The secondary endpoints include major cardiac adverse events, target vessel failure, stent thrombosis and angiographic efficacy endpoints.

**Discussion:**

The ECO-PLEASANT trial is the study not yet performed to directly compare the efficacy and safety of the Coroflex Please™ versus Taxus Liberte™ stent. On the basis of this trial, we will be able to find out whether the Coroflex Please™ stent is non-inferior to Taxus Liberte™ stent or not.

**Trial registration:**

ClinicalTrials.gov number, NCT00699543.

## Background

Previous randomized trials have shown the efficacy of a slow-release polymeric sirolimus-eluting stent (Cypher™, Cordis, Warren, NJ, USA), paclitaxel-eluting stent (Taxus™, Boston Scientific, Natick, MA, USA), and zotarolimus-eluting stent (Endeavor™, Medtronic, Minneapolis, MN, USA) over bare metal stents in reducing neointimal hyperplasia, late luminal loss, and angiographic restenosis leading to decreased target lesion revascularization [1-11] The Paclitaxel-eluting Coroflex Please™ stent is a newly developed drug eluting stent using the Coroflex^® ^stent platform combined with the drug paclitaxel contained in a polymer coating[12]

In the PECOPS I, which was one-arm observational study, the results of Coroflex Please™ stent were within the range of other Paclitaxel-eluting coronary stents [12,13] Compared with binary restenosis rate of 7.9% in Taxus IV trial, Coroflex™ Please stent showed 7.8% of restenosis rate[7] The 3.1% of 30 day MACE rate is within the range of other trials with stents eluting Paclitaxel or Sirolimus. The 6 month MACE rates in PECOPS I were 8.0%, which was similar to 7.8%, and 8.5% in Taxus II MR and SR, respectively[6] In Taxus IV, 9 month follow up revealed a MACE rate of 8.5%[7] In Taxus ATLAS trial, Taxus Liberte™ stent was proved to be non-inferior to TAXUS Express™ with 9 month TVR 8.0%[14] (Table 1) In other words, the efficacy of Coroflex Please stent has not been evaluated in wide range of lesion complexity met in the real world and not been compared with the similar taxol-eluting stents like Taxus stent.

**Table 1:** Angiographic characteristics of Studies with Paclitaxel-Eluting Coronary stents

	**Taxus IV** **(N = 662)**	**ATLAS** **(N = 871)**	**PECOPS I** **(N = 97)**
Reference, mm	2.75	2.75	2.89
MACE % (6-9 months)	8.5	11	8
TVR % (6-9 months)	4.7	8	-
TLR % (6-9 months)	3	5.7	5.7
Binary angiographic restenosis, %			
In-stent	5.5	11.4	3.9
In-segment	7.9	14.3	7.8
Late loss, mm			
In-stent	0.39	0.41	0.47
In-segment	0.23	0.25	-

Therefore, this study will compare the effectiveness and safety of the Coroflex Please™ stent versus the newest version Taxus Liberte™stent in patients with coronary disease including several lesion types that were excluded in previous clinical trial of PECOPS I to evaluate its efficacy and safety on relatively less-selected and more-"real-world" lesions.

### Study Objectives

The primary objective of this study is to evaluate the clinical efficacy, angiographic outcomes and safety of coronary stenting with the Coroflex Please™ stent system (B Braun, Germany), compared with the Taxus Liberte™ stent system (Boston Scientific, USA) in the treatment of coronary stenosis.

## Methods

### Study Design

This trial will be a prospective, randomized, open label, multi-center trial to demonstrate the non-inferiority of Coroflex Please™ stent compared with Taxus Liberte™ stent in "real world" coronary lesions.

#### Patient Enrollment

915 patients will be enrolled at 17 centers in Korea. Following angiography, patients with significant diameter stenosis >70% by visual estimation or patients with diameter stenosis >50% by visual estimation who have documented myocardial ischemia or symptoms of angina, and have lesions that are eligible for stenting without any exclusion criteria, will be randomized 2:1 to either a) Coroflex Please™ stent or b) Taxus Liberte™stent.

#### Patient Follow-up

Clinical follow-up will be at 1, 4, 9, 12 months and 2, 3 years after intervention. The investigator may conduct follow-up as telephone contacts or office visits. An angiographic follow-up will be required at 9 months to determine late luminal loss. Late angiographic follow-up will be recommended at 18-24 months post PCI in patients enrolled at selected centers.

### Endpoints

The primary efficacy endpoint is clinically driven Target Vessel Revascularization (TVR) at 9 months.

Clinical safety and efficacy secondary endpoints are Major Cardiac Adverse Events (MACE; all death, cardiac death, myocardial infarction (Q-wave and non-Q wave), TVR), Target Vessel Failure (TVF; cardiovascular death, myocardial infarction, clinically driven TVR) and stent thrombosis.

Angiographic secondary endpoints are in-stent binary restenosis by QCA; in-stent and in-lesion late loss by QCA; in-stent and in-lesion MLD and percentage diameter stenosis by QCA immediately after the index procedure and at follow-up.

### Patient Population

Total 915 patients derived from a population of Korean patients receiving percutaneous coronary intervention for ischemic heart disease will be enrolled in the present trial. Consecutive patients presenting at participating centers will be evaluated for the entry into the study. All consecutive patients (≥ 18 years of age) with coronary artery stenosis >50% by visual estimation will be screened for enrollment in this study and, if PCI is planned, should be invited to participate in the study.

Patients ≥ 18 years will be included in this study if they meet all of the following criteria: coronary artery stenosis (>50% by visual estimate) with evidence of myocardial ischemia (e.g., stable, unstable angina, myocardial infarction, silent ischemia, positive functional study or a reversible changes in the electrocardiogram (ECG) consistent with ischemia.) or significant coronary artery stenosis (>70% by visual estimate); Target lesion(s) located in a native coronary artery with visually estimated diameter of ≥ 2.5 mm and ≤ 4.0 mm; Target lesion(s) amenable for percutaneous coronary intervention.

This study excludes patents with followings: hypersensitivity or contraindication to the medications (heparin, aspirin, both clopidogrel and ticlopidine, paclitaxel, stainless steel or contrast media); Systemic (intravenous) Paclitaxel use within 12 months; Female of childbearing potential, unless a recent pregnancy test is negative, who possibly plan to become pregnant any time after enrollment into this study; History of bleeding diathesis or known coagulopathy (including heparin-induced thrombocytopenia), or willing to refuse blood transfusions; Gastrointestinal or genitourinary bleeding within the prior 3 months, or major surgery within 2 months; An elective surgical procedure is planned that would necessitate interruption of thienopyridines during the first 9 months post enrollment; Non-cardiac co-morbid conditions are present with life expectancy <1 year or that may result in protocol non-compliance (per site investigator's medical judgment); Patients who are actively participating in another drug or device investigational study, which have not completed the primary endpoint follow-up period; Patients with LV ejection fraction <25% or those with cardiogenic shock; Patients with acute ST elevation myocardial infarction who requires primary PCI; Patients with acute ST elevation myocardial infarction within 48 hrs; Creatinine level ≥ 3.0 mg/dL or dependence on dialysis; Severe hepatic dysfunction (AST and ALT: 3 times upper normal reference values); significant left main stem stenosis which requires revascularization therapy; target lesion has in-stent restenosis at the stented segment of drug-eluting stents or bare metal stents; Target lesions with bifurcating disease which require side branch stenting.

### Conduct of the study

After the patient has been enrolled in the present study, the following procedures will take place. The schedule of events for this trial is appeared in table 2. The treatment strategy will be determined by the study-certified interventional operator. It is recommended that each enrolling investigator review the most recently updated instructions for use (IFU) and assess the contraindications, warnings, and precaution sections for treating potential patients.

**Table 2:** Schedule of Events

	**Baseline**	**Post-Procedure**	**Follow up**			
			
			**30 days**	**4 mo**	**9 mo**	**Yearly**
			**±2 w**	**± 1 m**	**± 3 m**	**Up to 3 yrs**
Medical/Clinical History	**X**					

Informed consent	**X**^1^					

Inclusion/Exlusion Criteria	**X**					

Brief Physical Examination	**X**					

Vital status	**X**					

Weight, height	**X**					

12 lead ECG	**X**^4^				**X**	

Angiogram	**X**	**X**			**X**^5^	

CBC	**X**					

Electrolytes, LFT, Creatinine, BUN	**X**					

hs-CRP	**X**					

Fasting plasma triglycerides, HDL, total cholesterol	**X**					

Fasting glucose level^2^	**X**					

HgbA1C^3^	**X**					

Pregnancy test (if applicable)	**X**					

Medications	**X**		**X**	**X**	**X**	**X**

CK, CK-MB, Troponin I^6^	**X**	**X**				

Pro-BNP or BNP	**X**					

Event monitoring	**X**	**X**	**X**	**X**	**X**	**X**

^1^The informed consent may be signed either prior to the diagnostic angiogram or after the diagnostic angiogram.

^2^It may be done later, before discharge when the patient is in a fasting state

^3^In patients with diagnosed diabetes mellitus.

^4^Additional ECGs will be performed at 60 ± 30 minutes post-procedure. If the patient develops recurrent chest pain, ischemia, or significant arrhythmias, heart failure or other signs or symptoms of clinical instability, additional ECGs should be obtained

^5^Routine follow up angiography will be recommended at 9 months, but it can be performed at 9 months ± 3 months. Unscheduled angiograms > 6 months after index procedure will be considered as 9-month follow-up angiogram in final analysis.

^6^Optional in selected centers and if baseline lab is done, enzymes must be followed every 8-hours for 24 hours post-index procedure.

#### Index PCI

After random assignment to Coroflex Please™ or Taxus Liberte™ stents, the index PCI procedure must be carried out in all cases with in 7 days. Staged procedures carried out within one week will not be considered as repeat procedures. The goals of the procedure are to achieve optimal angiographic efficacy of PCI with allocated DES in selected target lesion sites while minimizing the risk of procedure-related complications. A study-certified interventional operator should perform all PCI procedures. An experienced catheterization laboratory staff should assist, and backup cardiac surgical support must be available on-site. The procedure should be performed in a cardiac catheterization laboratory that is capable of providing high quality digital images. A full range of commercially available guiding catheters, balloon catheters, and guide wires should be readily available. PCI may be performed by the radial or femoral approach.

Each procedure is preceded by a coronary angiogram of the vessels to be treated (diagnostic angiogram). At least 2 projections of each vessel should be obtained in orthogonal views. For each patient, a hierarchy of lesion priority is established such that PCI with drug-eluting stent implantation is attempted first in lesions that are most likely to be responsible for the patient's ischemia. In targeted lesions treated with stent implantation, the final angiographic objective is a <30% residual stenosis, whereas, in lesions treated with balloon PTCA (< 2.5 mm diameter vessels or unable to deliver the stent), the final angiographic objective is a <50% residual stenosis. In all treated vessels, necessary means should be taken to achieve TIMI grade-3 distal flow.

Paclitaxel-eluting stents will be used to treat all lesions suitable for drug-eluting stent placement. A patient should be treated with the same allocated stents in all lesions during the course of the trial. The placement of other than the allocated stent in a patient during the course of the trial will be accepted when the allocated stent cannot be deployed. If proper pre-dilation of the vessel has been done but the stent still fails to reach the lesion, the other type of study stent (ex, Coroflex Please™stent in case of Taxus Liberte™ stent delivery failure, or vice versa) may be considered. If both study stents cannot be delivered to the target lesion site, other commercially approved drug-eluting stent or bare stent or balloon PTCA may be used to complete optimal PCI. If the non-target vessel is too large (>4.5 mm) to be stented with allocated DES, bare-metal stent can be accepted. The length and diameter of stent will not be restricted.

##### Recommendations for the adjunctive pharmacological therapy

Aspirin in dose 300 mg PO must be administered before the index PCI, whether or not patient was taking Aspirin at home. Aspirin will be continued at 75-325 mg PO indefinitely. It will be recommended that patients receive oral 300 mg to 600 mg loading dose of clopidogrel before the index PCI if the patient was not taking clopidogrel within 24 hours prior to admission. Post-procedure, the treatment should be continued 75-150 mg PO per day at least for 9 months. In case of intolerance/allergy to clopidogrel, a loading dose of 500 mg of ticlopidine PO may be administered, and the treatment should be continued 250 mg Ticlopidine BID per day. The use of additional antiplatelet combination (i.e., cilostazol) will not be allowed.

#### Follow up

Clinical follow-up will be at the planned time points (Table 3). Follow-ups should be office visits, but telephone contact will be allowed. Data collected during all follow-up visits will include angina class and major adverse ischemic, neurologic and bleeding events, including re-hospitalization, re-catheterization and adverse events/serious adverse events. Original source documents must be submitted for any clinical events (death, re-infarction, revascularization, stroke, or any other SAE within 12 months). If the patient is readmitted to a non-study hospital, all possible efforts should be made to obtain original source documents from that hospital. For all re-infarctions, ECGs and cardiac enzymes (CPK, CK-MB, troponin) must be obtained and recorded.

**Table 3:** Clinical follow-up

**Follow-up time point**	**± days**
1 month	14

4 months	30

9 months	3 months

12 months	30

2 years	30

3 years	30

Routine angiographic follow-up at 9 months (-3 month/+3 months) will be recommended in this study. Any earlier angiogram >30 days showing restenosis or thrombosis (diameter stenosis >50%) will qualify as an endpoint angiogram. If an angiogram is performed between 1 and 4 months and restenosis is not present in any study lesion, the requirement for the 9-month angiogram has not been met and thus the 9-month angiographic follow-up must still be performed. Even when there was unexpected angiography between 4 and 6 months and restenosis or thrombosis were not present in any study lesion, the 9-month angiographic follow-up must still be performed. However, unscheduled angiograms > 6 months after procedure will be considered as 9-month follow-up angiogram in final analysis. Copies of angiograms must be submitted to the angiographic core laboratory of Seoul National University Hospital Cardiovascular Center. Angiograms to be received by the core laboratory include:

• The baseline angiogram from all randomized patients;

• The 9-month routine follow-up angiograms in the follow-up;

• All unscheduled follow-up angiograms in all randomized patients over the 1 year period.

• Late angiographic follow-up will be recommended at 18-24 months post PCI in patients enrolled at selected centers.

### Statistical Considerations

#### Sample size calculation

To test the hypothesis that Coroflex Please™ stent is non-inferior to Taxus Liberte™ stent in 'clinically driven TVR at 9 months', we assumed an incidence of TVR as 8% for the Taxus Liberte™ stent and less than 5% increases for the Coroflex Please™ stent on the basis of results from TAXUS II, TAXUS IV, ATLAS and PECOPS I trials with type I error set at 0.05, statistical power set at 80%, sampling ratio of Coroflex Please™ stent: Taxus Liberte™ stent at 2:1, and an estimated drop out rate of 10% (for 9-month clinical follow up). Based on above assumptions, we would need a total of 915 patients, 610 patients in Coroflex Please™ arm and 305 in Taxus Liberte™ arm.

To test the hypothesis that Coroflex™ Please stent is non-inferior to Taxus Liberte™ stent in inhibiting 'neointimal growth at 9 months angiographically', we assumed the late loss as 0.4 ± 0.5 mm for the Taxus Liberte™ stent and less than 0.15 mm increases for the Coroflex™ Please stent on the basis of results from TAXUS II, TAXUS IV, ATLAS and PECOPS I trials with type I error set at 0.05, statistical power set at 80%, sampling ratio of Coroflex Please™ stent: Taxus Liberte™ stent at 2:1, and an estimated drop out rate of 30% (for 9-month follow up angiography) [6,7,12,14]. Based on above assumptions, we would need a total of 450 patients, 300 patients in Coroflex Please™ arm and 150 in Taxus Liberte™ arm. Finally, based on above calculations, we would need a total of 915 patients, 610 patients in Coroflex Please™ arm and 305 in Taxus Liberte™ arm.

#### Statistical analyses

All primary and secondary endpoints will be analyzed both on an intention-to-treat basis (all patients analyzed as part of their assigned treatment group) and on per protocol basis (patients analyzed as part of their assigned treatment group only if they actually received their assigned treatment). For intention-to-treat analysis, all patients who signed the written informed consent form and are randomized in the study will be included in the analysis sample, regardless of whether or not the correct treatment was administered, or whether crossover occurred. For the per protocol analysis, only enrolled patients who actually received the assigned treatment will be included in the analysis sample.

Baseline characteristics of study patients will be summarized in terms of frequencies and percentages for categorical variables and by means with standard deviations for continuous variables. Categorical variables will be compared by Fisher's exact test. Continuous variables will be compared by the 2-sample *t *test. Cumulative event-free survival will be summarized as Kaplan-Meier estimates. A p value of 0.05 will be established as the level of statistical significance for all tests.

The primary as well as secondary endpoints will be analyzed in prespecified subgroups. These subgroups include patients with diabetes mellitus and long lesions.

### Trial Organization

#### Executive Committee

The Executive Committee will be composed of the study chairperson and the principal investigators of the investigating centers. This committee will approve the final trial design and protocol issued to the Data and Safety Monitoring Board (DSMB) and the clinical sites. This committee will also be responsible for reviewing the final results, determining the methods of presentation and publication, and selection of secondary projects and publications by members of the Steering Committee.

#### Data Safety Monitoring Board

The DSMB is composed of general and interventional cardiologists, and a biostatistician. Names of the actual members will not be announced, but may be provided to the regulatory agency upon request. The DSMB will function in accordance with applicable regulatory guidelines. The board members are independent and will not be participating in the trial. The DSMB committee will review the safety data from this study and make recommendations based on safety analyses of unanticipated device effects (UADEs), serious adverse events (SAEs), protocol deviation, device failures, and 30-day follow-up reports. The frequency of the DSMB meetings will be determined prior to study commencement. Additionally, the DSMB may call a meeting at any time if there is reason to suspect that safety is an issue. The DSMB is responsible for making recommendations regarding any safety or compliance issues throughout the course of the study and may recommend the Executive Committee to modify or stop the study. However, all final decisions regarding study modifications rest with the Executive Committee.

All cumulative safety data will be reported to the DSMB and reviewed on an ongoing basis throughout enrollment and follow-up periods to ensure patient safety. Every effort will be made to allow the DSMB to conduct an unbiased review of patient safety information. All DSMB reports will be made available to the appropriate agencies upon request but will otherwise remain strictly confidential.

Prior to the DSMB's first review of the data, the DSMB charter will be drafted. The plan will define the stopping rules for stopping the trial for safety. The first meeting of the DSMB will be sued for discussion of the protocol and an understanding of all the protocol elements. The DSMB will develop a consensus understanding of all trial endpoints and definitions used in the event adjudication process. All DSMB reports will remain strictly confidential, but will be made available to the regulatory body upon request.

#### Clinical Event Adjudication Committee

The Clinical Events Adjudication Committee (CEAC) is comprised of interventional and non-interventional cardiologists who are not participants in the study. The CEAC is charged with the development of specific criteria used for the categorization of clinical events and clinical endpoints in the study which are based on protocol. At the onset of the trial, the CEAC will establish explicit rules outlining the minimum amount of date required, and the algorithm followed in order to classify a clinical event. All members of the CEAC will be blinded to the primary results of the trial.

The CEAC will meet regularly to review and adjudicate all clinical events in which the required minimum data is available. The Committee will also review and rule on all deaths that occur throughout the trial.

#### Data Coordination and Site Management

Data coordination and site management services will be performed by the Clinical Trials Center at Seoul National University Hospital.

### Ethical approval

This study has been approved by institutional review board of Seoul National University Hospital.

## Discussion

Drug-eluting stent (DES) has revolutionized the field of interventional cardiology. Before DES era, restenosis in the stented lesion was problematic. However, DES has significantly reduced the rate of restenosis from 20-30% to single digits as compared with bare-metal stents by inhibiting the growth of neointima after stenting[1,2,7] As a consequence, nowadays interventional cardiologists are increasingly treating patients with tougher and tougher lesion, who would have otherwise undergone coronary artery bypass surgery.

Paclitaxel, a liphophilic component from the Pacific yew tree *Taxus brevifolia*, stabilizes the assembly of microtubules by binding β-tubulin dimers and inhibiting their polymerization [15-17] This drug inhibits arterial smooth muscle cell proliferation and migration and matrix proliferation resulting in inhibition of neointimal hyperplasia after coronary artery stenting[18,19] Taxus is the first Paclitaxel-eluting stent approved for human use. Its efficacy and safety have been proven in numerous randomized prospective trials including the TAXUS II, TAXUS IV and TAXUS VI trials [6-9]

In TAXUS II trial, Taxus slow-release (SR) and Taxus moderate-release (MR) showed a significant benefit over the bare metal stent (BMS). Compared with late loss of 0.79 ± 0.45 mm and 0.77 ± 0.50 mm in the respective BMS control groups, Taxus SR and MR reduced late loss by 60.7% and 61.0%, respectively (0.31 ± 0.38 mm and 0.30 ± 0.39 mm). Also, compared with percent in-stent net volume obstruction of 23.17 ± 18.19% and 20.54 ± 16.68% in the respective control groups, Taxus SR and MR showed a significant favorable result of 7.84% ± 9.87% and 7.84 ± 9.66%. In terms of clinical efficacy as estimated by MACE at 12 months, Taxus SR and MR showed 10.9% and 9.9%, compared with 22.0% and 21.4% in the respective control BMS groups.

Taxus IV trial was a prospective, randomized, double-blind study in which 1314 patients were enrolled. The rate of ischemia-driven target-vessel revascularization at nine months was reduced from 12.0% in BMS group to 4.7% in Taxus SR group. Target-lesion revascularization was required in 3.0% of Taxus SR group, as compared with 11.3% of BMS group. Moreover, the rate of angiographic restenosis was reduced from 26.6% in BMS group to 7.9% in Taxus SR group.

In Taxus VI trial which was a prospective double-blind, randomized trials assessing clinical and angiographic outcomes of Taxus MR in the treatment of long, complex coronary artery lesions, target-vessel revascularization rate at 9 months was 9.1% in the Taxus group and 19.4% in BMS group. Target-lesion revascularization was reduced from 18.9% in the BMS group to 6.8% in the Taxus group. The incidence of major adverse cardiac events was similar in the 2 groups. Angiographically, in-stent late loss was reduced 0.99 ± 0.585 mm to 0.39 ± 0.560 mm.

In Taxus ATLAS trial which was a global, prospective, single-arm trial assessing non-inferiority of Taxus Liberte™ versus Taxus Express™ target-vessel revascularization rate at 9 months was 8.0% in Taxus Liberte™ group and 7.1% in Taxus Express™ group. Major adverse cardiac events rate at 9 months was 10.5% and 11.0% respectively. In terms of angiographic outcomes, in-stent late loss was 0.41 ± 0.54 mm versus 0.42 ± 0.54 mm.

The highly flexible Paclitaxel-eluting Coroflex Please stent is a recently developed DES[13] This features stainless steel struts covered by the non-absorbable polymer Polysulfone^® ^which is thermostable upto 180°C and thus does not lose its physical properties during sterilization, transportation, nor storage. The release kinetics of the drug ranges between Taxus SR and MR.

PECOPS I trial, which enrolled 97 patients, was multicenter, prospective, nonrandomized trial to evaluate procedural, angiographic and clinical results of Coroflex Please™ stent[12,13] The success rates of procedure and study stent deployment were 100% and 94.8%. Binary in-segment restenosis rate at 9 months was 7.8% as compared with 7.9% in TAXUS IV trial. At 6 months, the MACE rate was 8.0%, which was 8.5% and 7.8% for TAXUS II MR and SR, respectively. These results of Coroflex Please™ stent are within the range of other trials with other Paclitaxel-eluting coronary stents. However, PECOPS I was one-arm observational study and the lesion more than 16 mm in length was excluded. Hence, we cannot infer that Coroflex Please™ has comparable clinical outcome for longer lesion. Furthermore, there have been no previous head-to-head comparisons between Coroflex™ Please and Taxus both of which elute paclitaxel.

In the current ECO-PLEASANT trial, we plan to directly compare the angiographic and clinical efficacy of Coroflex Please™ versus Taxus Liberte™ in coronary lesion including long one. Moreover, we will compare the efficacy in diabetic patients. We will mandate a 9-month follow up coronary angiogram to compare the late luminal loss between the two types of stents. Oculostenotic reflex may become a confounding variable to re-intervention rate since we expect a 9-month angiographic follow up of over 70%. To reduce the chance of falsely high rate of repeat intervention, we will strictly differentiate clinically-driven revascularization from oculostenotic reflex-driven repeat intervention. In addition, all events will be independently and judiciously adjudicated by the clinical event adjudication committee.

Recently 'late-catch up' phenomenon emerges as the hot issue of DES. In animal studies, DES-treated segments showed the delayed neointimal hyperplasia, which was not definite in BMS-treated ones[20,21] Carter et al. found that sirolimus-eluting stents did not maintain long-term inhibition of neointimal hyperplasia because of the polymer-induced inflammation or the delayed cellular proliferation in the porcine coronary model[20] Farb et al. showed that paclitaxel-eluting stents gave rise to delayed healing and local toxicity which might be associated with delayed neointimal hyperplasia[21] Park et al. demonstrated that despite the 6-month suppression of intimal hyperplasia after paclitaxel-eluting stents compared with BMS, there was a "late-catch up" of intimal growth during subsequent 18 months[22] Thus in this ECO-PLEASANT trial, we will mandate an 18-24 months follow up coronary angiogram at selected centers to answer the question; "Is there late catch-up phenomenon in Coroflex™ Please or Taxus Liberte™ stenting?"

In conclusion, this ECO-PLEASANT study is the first randomized controlled trial of Coroflex Please™ stent, and it will give us, for the first time, the insight regarding the comparative efficacy of Coroflex Please™ stent with Taxus Liberte™ stent, both of which do not have data in the situation of randomized controlled trial. Furthermore, we include lesions with wide range of complexity in this trial, which will give us valuable efficacy data easily extrapolatable to the real world practice. Finally, by adopting 18-24 months follow up coronary angiography in addition to 9 months one, we may be able to get the precious information regarding 'late catch up phenomenon' in these stents.

## Abbreviations

ECO-PLEASANT trial: comparison of Efficacy between COroflex PLEASe ANd Taxus™ stent trial; PES: Paclitaxel-Eluting Stent; MACE: Major Adverse Cardiac Events; PCI: Percutaneous Coronary Intervention; TVR: Target Vessel Revascularization; TVF: Target Vessel Failure; QCA: Quantitative Coronary Angiography; MLD: Minimal Luminal Diameter; DES: Drug-Eluting Stent; PTCA: Percutaneous Transluminal Coronary Angioplasty; DSMB: Data and Safety Monitoring Board; UADEs: UnAnticipated Device Effects; SAEs: Serious Adverse Events; CEAC: Clinical Events Committee; BMS: Bare Metal Stent.

## Competing interests

The authors declare that they have no competing interests.

## Authors' contributions

HSK is the PI of this study and KWP developed the trial protocol. JBS is the study coordinator. KWP, HKJ, JSP, JHB, SWK, KWM, JWC, SGL, WYC, TJY, SJK, DIK, BOK, MSH, KSP, TJC, HKH and SHH all managed the project at each hospital or medical center. All authors have read and approved the submission of this manuscript.
